# Sensitivity of Beech Trees to Global Environmental Changes at Most North-Eastern Latitude of Their Occurrence in Europe

**DOI:** 10.1100/2012/743926

**Published:** 2012-05-02

**Authors:** Algirdas Augustaitis, Dalia Jasineviciene, Rasele Girgzdiene, Almantas Kliucius, Vitas Marozas

**Affiliations:** ^1^Faculty of Forestry and Ecology, Aleksandras Stulginskis University, Akademija, 53362, Kaunas District, Lithuania; ^2^Environmental Physics and Chemistry Laboratory, Center for Physical Sciences and Technology, 02300 Vilnius, Lithuania

## Abstract

The present study aimed to detect sensitivity of beech trees (*Fagus sylvatica* L.) to meteorological parameters and air pollution by acidifying species as well as to surface ozone outside their north-eastern distribution range. Data set since 1981 of Preila EMEP station enabled to establish that hot Summers, cold dormant, and dry and cold first-half of vegetation periods resulted in beech tree growth reduction. These meteorological parameters explained 57% variation in beech tree ring widths. Acidifying species had no significant effect on beech tree growth. Only ozone was among key factors contributing to beech stand productivity. Phytotoxic effect of this pollutant increased explanation rate of beech tree ring variation by 18%, that is, up to 75%. However, due to climate changes the warmer dormant periods alone are not the basis ensuring favourable conditions for beech tree growth. Increase in air temperature in June-August and decrease in precipitation amount in the first half of vegetation period should result in beech tree radial increment reduction. Despite the fact that phytotoxic effect of surface ozone should not increase due to stabilization in its concentration, it is rather problematic to expect better environmental conditions for beech tree growth at northern latitude of their pervasion.

## 1. Introduction

European beech (*Fagus sylvatica *L.) is one of the most widespread and important tree species in Europe due to its high physiological tolerance to the abiotic and biotic threats accompanying climate change [1, 2]. Its proportion is currently increasing in Central Europe, particularly where forests with a high percentage of conifers are being converted into mixed forests [3, 4]. Increased competitiveness compared to boreal tree species ensures recent northward migration of European beech, which resulted in arising new issues in areas outside their natural distribution range in north-eastern part of Europe, where the winters became warmer than before [5].

It is well established that changes in tree condition, in most cases, are a result of the multitude of stress factors including environmental pollution, and rapidly changing climatic conditions, that is, heat and drought over the vegetation period as well as frost over the dormant period, where air concentrations of acidifying species and their deposition play a predisposing, accompanying and locally, even a triggering role [6–12]. Ozone as accompanying agent of climate changes is also among key factors resulting in spatial and temporal changes of tree crown condition and productivity [13–18].

The findings of our earlier study allowed us to make an assumption that temporal and spatial changes in crown defoliation and stem radial increment of the prevailing in Lithuania Scots pine trees are first of all, related to air concentrations of the acidifying compounds and their deposition as well as to meteorological parameters which can reinforce or mitigate the integrated impact of these factors [19–21]. In this study, we attempted to investigate possible effect of natural and anthropogenic environmental factors on stem radial growth of alien tree species in Lithuania-European beech.

The variation in tree ring width of European beech was found to be a very sensitive indicator, reflecting clearly the signals of environmental influences [22]. One of the first symptoms expressed by declining trees is reduced radial increment of their stems [10, 23]. These changes are more conspicuous than the growth of coniferous trees [24]. Therefore, the present study aimed to detect sensitivity of beech tree growth to natural and anthropogenic environmental factors and quantify O_3_ contributions to the integrated impact of these factors in Lithuania by using dendrochronological and multiple regression methods. The objectives of the study were

- to detect the effect of meteorological parameters on beech tree stem growth;
- to detect key air pollutants limiting beech tree growth;
- to quantify O_3_ contributions to the integrated impact of the considered environmental factors on beech trees growth;
- to prognose growth peculiarities of beech trees in Lithuania under rapidly changing environmental conditions.

The obtained results allowed us to test the hypothesis that north-eastern part of Europe due to warmer dormant and more humid vegetation periods became more favorable for beech tree growth even in the areas outside beech natural distribution range.

## 2. Materials and Methods

### 2.1. Study Sites and Their Location

**Figure 1:**
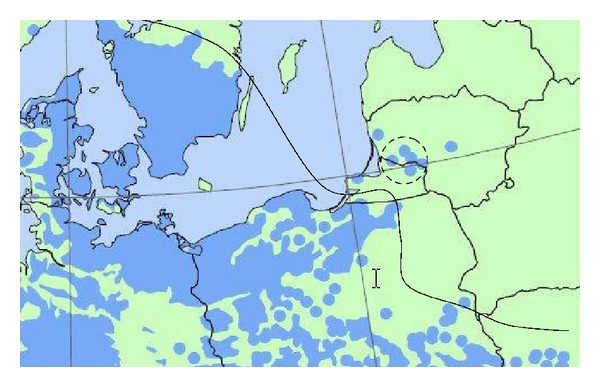
The edge of beech tree distribution range in north-eastern part of Europe (continuous line) and location of experimental sites (dotted line) EUFORGEN [28].

Beech is considered to be a climax species in most parts of Europe, where it grows in very different environments. Beech is a monoecious, anemophilous, and mostly allogamic species [25]. Pure and mixed beech stands cover more than 12 million hectares, from southern Scandinavia to the Iberian, Italian and Balkan Peninsulas, and from southern England to Ukraine [26]. In Central Europe, beech is a component of lowland forests, while in the Mediterranean area it is a typical mountain species [27]. In Lithuania, beech stands were planted by Prussian foresters in the nineteenth century. Four monitored beech stands were located at about 150 km north from the edge of their natural distribution range in Poland (Figure 1). About 100 tree ring width series were used to meet the objectives of the study.

### 2.2. Dendrometric Parameters

Data on tree and stand parameters (mean diameter and height of trees, tree density, basal area, and tree volume per hectare) were available from the circular permanent observation plots (CPOPs) radius of which made 17.85 m and area 1000 m^2^. During the field work, the location of each monitored tree was detected by measuring the azimuth and distance from the plot centre. Stem diameter and crown defoliation for all sample trees were measured and assessed until September at the latest, due to which the considered response variables (meteorology and pollutants) were analysed over the period from September of the previous year to August of the current year. Main dendrometric characteristics of the monitored stands are compiled in Table 1.

In the monitored stands beech grows mainly with Scots, pine or Oak trees in the second story increasing stand volume by approximately 200 m^3^ until 600–800 m^3^ which makes about 30–40% of total stand volume. The age of beech trees do not exceed 70 years. Only in the first monitored stand B1, beech tree grows in the first story after the Scots pine trees were cut at the beginning of 2000.

Crown defoliation of the trees was monitored from the end of July up to beginning of September employing European forest monitoring methodology [29]. Mean annual values of crown defoliation as well as stem diameter were computed for every CPOP. Every third monitored tree was selected for more detailed measurements of the tree crown parameters, height, and tree ring analysis. Each ring was measured to the closest 0.01 mm using an electronic transducer and binocular scope fixed over the moving stage.

A standard dendrochronological technique was used to assess tree growth rates. To eliminate effect of age tree ring width indices were computed using formula:

(1) $$Zr_{\text{index}}=Zr-\left(a\;+\;b\;\times \;\text{Year}\right),$$

here: *Zr* index: ring index in the year (i); *Zr*: tree ring width (mm); Year: year from 1960 to 2010; *a*, *b*: regression coefficients.

The linear, instead of curvilinear, regression was used with the aim of evaluating rapid changes in meteorology and pollutants which occurred by evidently expressed tendencies.

### 2.3. Main Predictor Variables and Methods of Their Estimation

#### 2.3.1. Air Pollution and Deposition

Data on air concentrations of sulphur dioxide (SO_2_), sulphate (SO_4_^2−^), and wet deposition of SO_4_^2−^, NO_3_^−^, and NH_4_^+^ as well as surface ozone concentrations since 1981 were used to detect sensitivity of beech trees outside their natural distribution range. ΣNO_3_^−^ and ΣNH_4_^+^ air concentrations have been monitored at the station only since 1994; therefore, this acidifying species was eliminated from the analysis.

Air sampling was carried out at weekly intervals over the entire year. The sampling equipment for SO_2_ and particulate sulphate consisted of a two-stage filter pack sampler with a cellulose filter (Whatman 40). SO_2_ was collected by retention of particles using Whatman 40 filter impregnated with potassium hydroxide (KOH).

Weekly precipitation samples were collected in a polyethylene bulk-collector from December to March (snow collector) and in an automatic wet-only sampler during the remaining months. All samples were stored at 4°C until laboratory analysis.

Ion chromatography using Dionex 2010i with conductivity detection was used for the chemical analysis of anions in precipitation and in water extracts from the impregnated Whatman 40 filters. The NH_4_^+^ concentration in precipitation as well as in the solutions extracted from Whatman 40 filters impregnated with oxalic acid was analysed spectrophotometrically, using the indophenol blue method.

Ozone concentrations were measured continuously using commercial UV-absorption ozone monitors O_3_ 41 M (Environnement S.A, France) and ML9811 (Monitor Labs) with an air inlet at the height of 2.5 m above ground. The instruments were calibrated against the reference standard UV photometer SRP11 at Stockholm University in Sweden from 2002 to the present, against the reference standard UV photometer SRP17 at the Czech Hydrometeorological Institute. Hourly data on peak ozone values, their annual average, and averages over the entire year-long period were used in the analysis. AOT40 values, which define the potential risk of O_3_ for vegetation [30], due to the lack of necessary data, were computed only since 1994 and, therefore, were not included into analysis.

The measurements and analytical procedures were based on a quality assurance/quality control (QA/QC) programme as described in the EMEP CCC manual for sampling, chemical analysis, and quality assessment [31].

#### 2.3.2. Meteorological Parameters

Time series of monthly temperature and precipitation were used to characterize climate change conditions at the sampling stands. The effect of meteorological conditions on beech trees growth was analysed by beginning in autumn of the previous year (in September) and ending in summer of the current year (in August). Meteorological data were obtained from the nearest to beech stands Šilute meteorological station, located about 15 km away from the monitored sites. The quality of the data was assured according to the requirements of the World Meteorological Organization [32].

### 2.4. Statistical Methods

In traditional dendroecology, tree growth is usually investigated by employing response function analysis of climatic factors [33–35] with significant correlations identification [36]. The average monthly temperatures and monthly precipitation are treated as growth-limiting factors [37, 38]. Based on significance of the detcted correlations between monthly meteorological parameters and tree ring indices, periods by aggregating data on monthly parameters were created, and their impact on beech growth was evaluated. Afterwards annual tree ring indices were regressed on aggregated meteorological parameters (air temperature and precipitation amount) using a regression of Statistica 6.0 software with a forward selection procedure to detect integrated effect of meteorological parameters on beech growth. The effect of pollutants on beech tree growth was examined in a second step of multiregression procedure [39] when the effect of aggregated meteorological parameters on beech tree ring indices was accounted for. The residual indices were regressed on air concentrations of acidifying compounds, their concentrations in precipitation and deposition as well as surface ozone using the same stepwise regression procedure. Finally, contributions of key pollutants to the integrated effect of meteorological parameters on beech tree ring indices were quantified. To assess the quality of the model, we used coefficient of determination *R* ^2^, when *P* < 0.05.

## 3. Results and Discussions

### 3.1. Radial Growth of Beech Tree Stems

**Figure 2:**
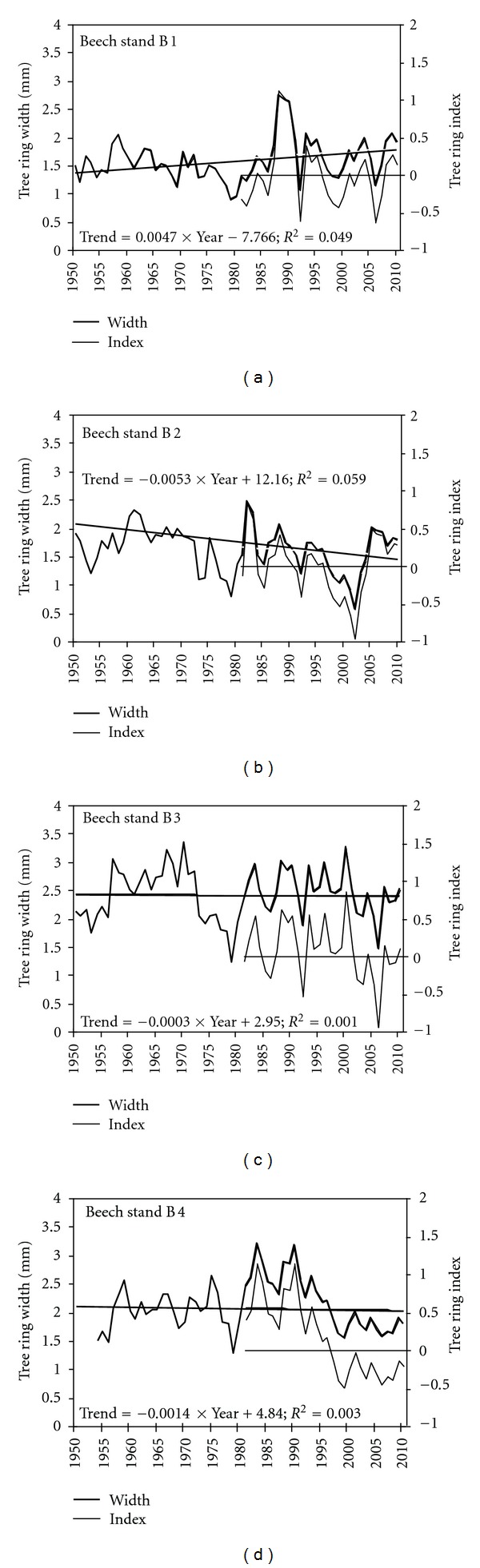
Mean tree ring with and their indices in monitored beech stands.

In Central Europe since 1950 at lower-altitude sites mainly increased growth trends have been obvious, whereas at higher altitude sites almost all sites have shown a slightly decreased growth potential especially during the last decades [22]. Analysis of tree ring widths showed that beech tree growth in general demonstrated no regular pattern (Figure 2).

Increase in radial increment was observed only in the 1st monitored beech stand B1, where beech trees grow in the first story. Between 1980 and 1990, increase in radial increment was registered in B4 stand; however, afterwards a significant decrease in tree ring width was obvious. Tree growth in stand B2 demonstrated a tendency towards reduction over the entire period, meanwhile in B3 stand radial increment was stable at the level near 2.5 mm. Minimum values of tree ring width were observed in 1979 and between 1997 and 2005. The most favorable period for beech growth was the beginning of 1980s (after a very cold winter in 1979) and over the last 5 years, that is, since 2005 up to now. These periods were taken into account analyzing variation in air temperature and precipitation amount. Obtained data did not confirm the state of knowledge about the increase in beech tree growth at lower altitude [22].

### 3.2. Meteorological Parameters, Their Changes, and Effect on Beech Tree Radial Increment

**Figure 3:**
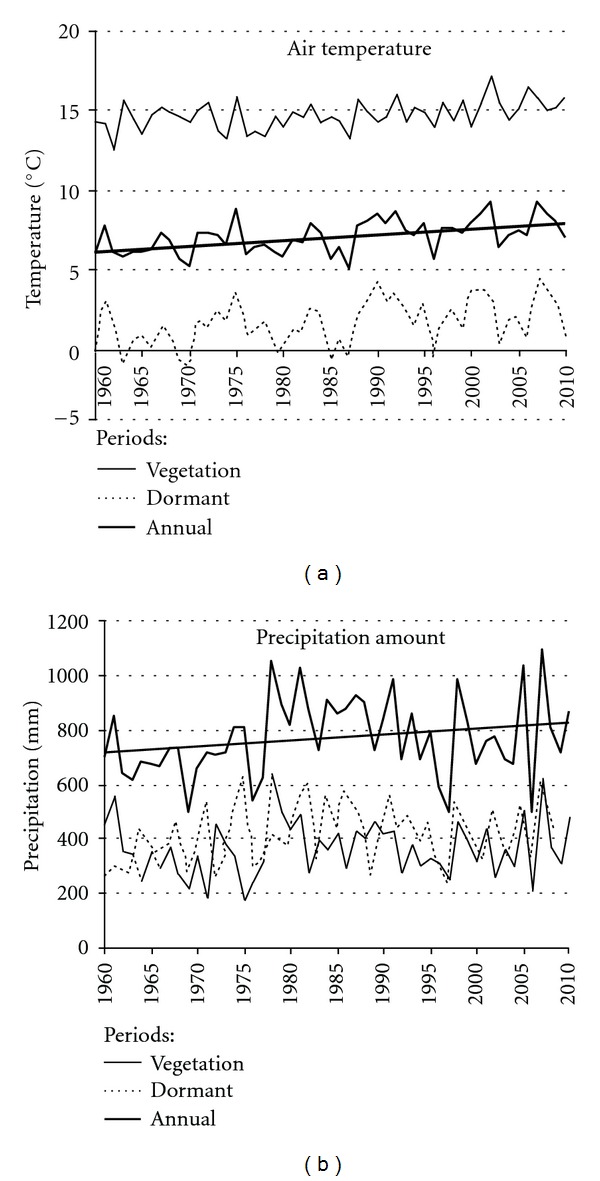
Changes in main meteorological parameters over the last 50-year period.

Changes in mean air temperature of the warmest, coldest months, and annual mean value indicated that the warmest years were 1961, 1967, 1975, 1983 1990–92, 2002, and 2007 (Figure 3) when mean air temperature of these year increased from +7.7°C up to 9.2°C (mean air temperature of the dormant period increased from +3.1 up to +4.5°C, mean air temperature of the vegetation period—from +14.1 up to +17.2°C). The coldest years were 1963, 1970, 1979-80, 1985–1987, 1996, and 2003, meanwhile their dormant period values demonstrated a tendency towards increasing too from −1.29 up to −0.27°C. Long-term variation in mean air temperature revealed that over the vegetation period air temperature demonstrated a tendency towards increasing by +0.027°C per year, over the dormant period by +0.042°C and annual value by 0.035°C per year which very well agreed with the data presented by the SRES A1 B Project (3.5°C per 100 years) [40].

Lack of humidity was registered in 1976-77, 1997, and 2006, when annual amount of precipitation did not exceed 600 mm value and made only about 30–40% from the optimum values of this tree species (1500 mm according to Teissier du Cros et al. [26]) (Figure 3). Years with excessive humidity were between 1978 and 1991, and also 1998, 2005, and 2007 when amount of precipitation exceeded 1000 mm which made about 60% of the optimum. Precipitation amount over the vegetation period demonstrated a tendency towards increasing by +0.7 mm, and over the dormant period by +1.5 mm. Consequently, annual amount increased by +2.2 mm per year. These changes were not statistically significant (*P* > 0.05) and differed from the optimum for beech tree growth essentially, what in turn had to result in specific reaction of beech trees growth.

Beech prefers oceanic climates (no extreme winter temperatures, cool and foggy Springs, and mild Summers) with mean annual rainfall higher than 1500 mm. Beech does not tolerate extreme water stress and grows better in deep and well-drained soils [26]. During the vegetation period, especially in June and July, low temperature and high precipitation support the formation of wide tree rings as well as warm, sunny and dry early Autumn promotes radial growth at the beginning of the following season [22].

**Figure 4:**
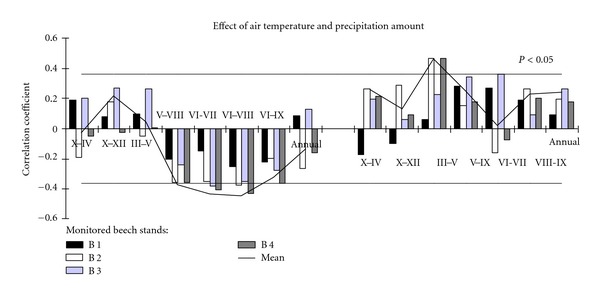
Effect of air temperature and precipitation amount on beech tree-ring indices in monitored stands over the 31 years period.

Performed correlative analysis of the relationships between meteorological parameters and beech tree radial increment over the period from 1981 up to 2010 revealed that warmer dormant periods, especially over October–December and cooler vegetation periods, especially over June–August resulted in wider rings of beech trees as well (Figure 4). More abundant precipitation over March–May followed by higher air temperature also resulted in wider beech stem ring. These data were in close agreement with many statements on key factors limiting beech growth in Central and Southern Europe [22, 26, 27]. However, negative effects of droughts in the middle of vegetation period on annual beech tree ring width [22] were not confirmed.

To generalize the obtained relationships of the integrated effect of meteorological parameters on beech tree growth the following multiple equation was implied:

(2) $$\begin{array}{cc} Zr_{\text{index}}\; & =4.61-0.177Tm_{\left(\mathrm{VI}-\text{VIII}\right)}-0.124\times {Tm^{\prime }}_{\left(\mathrm{VI}-\text{VIII}\right)}\\ & \;+\;0.021\times Tm_{\left(\text{X}-\text{XII}\right)}+0.0006\;\\ & \;\times \left[Tm\times {\text{Pr}}_{\left(\text{III}-\text{V}\right)}\right];\;R²=\;0.566;\;P<0.0002, \end{array}$$

where: *Tm*: mean air temperature of current year; *Tm*′: of previous year; Pr: precipitation amount;  *Tm* × Pr: multiplication of temperature and precipitation amount; (*in brackets*): the selected period in months.

Meteorological parameters explained 57% variation in tree ring width indices. Hot Summer, cold Winter and cold and dry first-half of vegetation period seem to be the main beech tree growth-limiting factors in Lithuania.

### 3.3. Acidifying Species, Their Deposition, Changes, and Effect on Beech Tree Radial Increment

**Figure 5:**
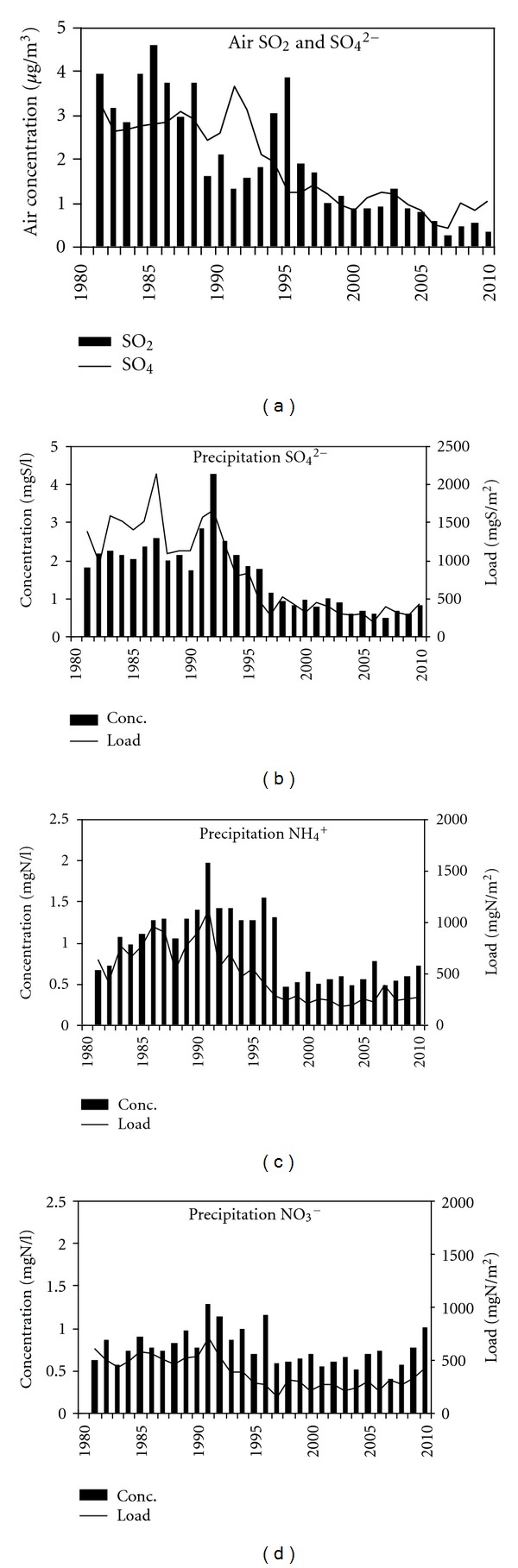
Main acidifying species in air and precipitation and their changes over the 30-year period.

Data of Preila EMEP station showed a significant decrease in concentrations of the main acidifying species in air as well as in precipitation until the year 2000. Over this period air concentrations of both sulphur compounds (SO_2_ and SO_4_^2−^) decreased by approximately 75–80% (SO_2_ from 4.0 to 0.85 *μ*gS/m^3^ and SO_4_^2−^ from 3.0 to 0.83 *μ*gS/m^3^). Sulphur concentration in precipitation and its wet deposition decreased by 70% and 85%, respectively, that is, concentration in precipitation from 2.5 mg/L to 0.8 mg/L and wet deposition from 2100 mg/m^2^ to 310 mg/m^2^ (Figure 5). This significant decrease (*P* < 0.05) in annual sulphur compounds was most likely the result of a reduction in SO_2_ emissions in Europe including Lithuania [41].

Data on NO_3_^−^ and NH_4_^+^ concentrations in precipitation and wet deposition revealed that the highest concentration of ammonia in precipitation and level of its wet deposition were reached in the period between 1987–1993 (1992 NH_4_^+^ 2.0 mgN/L and 1120 mgN/m^2^). Afterwards pollution level by this species decreased significantly up to 0.6 mgN/L and 200 mgN/m^2^ in 2000, which made 70% and 82%, respectively. Changes in NO_3_^−^ deposition were the least expressed, meanwhile some decrease in wet concentration and wet deposition was detected. Until 2000 NO_3_^−^ concentration in precipitation decreased by 53% (from 1.3 mgN/L to 0.6 mgN/L) and wet deposition by 72% (from 700 mgN/m^2^ to 200 mgN/m^2^).

Between 2000–2007, a further decrease in SO_2_ and aerosol SO_4_^2−^ air concentrations up to 0.26 *μ*gS/m^3^and 0.40 *μ*gS/m^3^ respectively was observed (Figure 5). Those were the least values of this acidifying species over the entire observation period. Afterwards, gradual increase in air concentration of these sulphur species started, and in the last 2010 year these values increased up to 0.31 *μ*gS/m^3^and 1.02 *μ*gS/m^3^ respectively. In SO_4_^2−^ wet concentrations and deposition data series, the same tendencies of variation were detected. The least values of SO_4_^2−^ concentration in precipitation over the last 2000—2010 period was detected in 2007, when it reached 0.46 mg/L and wet deposition in 2006—178 mg/m^2^. In the last 2010 year these values increased again up to 0.8 mg/L and 420 mg/m^2^.

Since 2000 NH_4_^+^ deposition and its concentration in precipitation, contrary to sulphur variation, started, increase gradually by 6.8 mg/m^2^ per year (*P* > 0.05) and 0.014 mg/L per year. In 2010 their values exceeded 0.7 mg/L and 260 mg/m^2^, respectively. Increase in NO_3_^−^ deposition and its concentration in precipitation was two times higher and made 12.8 mg/m^2^ and 0.027 mg/L per year. In 2010 NO_3_^−^ concentration in precipitation exceeded 1.0 mg/L and deposition 400 mg/m^2^ values, respectively. Detected peculiarities in variation of acidifying species were taken into account analyzing their possible effect on formation of the ring width of beech trees.

Negative effect of SO_2_ air concentration and S and N wet deposition on forest especially over high pollution periods is well documented [9, 42, 43] as well as in Lithuania [20, 44]. However the main research object of these studies was coniferous and mainly Scots pine. Much less evidence is presented about the effect of acidifying species on deciduous tree species and on beech tree in particular. Implementation of the Gothenburg Protocol and other legislation, which reduced emission of the acidifying species, reduced the possibility of forest health deterioration due to decreased acidification of forest ecosystem [45]. Despite this, in areas of beech trees natural distribution range data on beech tree growth reduction under the effect of elevated concentrations of acidifying species are presented [46, 47].

**Figure 6:**
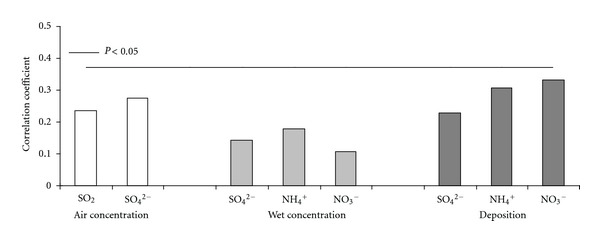
Effect of the main acidifying species in the air, precipitation, and their deposition on beech tree radial growth.

Obtained results on correlative analysis of the residual part of radial increment when the effect of meteorology was accounted for and acidifying species revealed the absence of significant relationships, that is, *P* > 0.5 (Figure 6).

The detected positive effect of the acidifying species on beech growth is explained first of all by coincidence of variation in beech tree ring width and acidifying species concentrations and secondly by very low air concentrations of S and N species as well as their deposition. Only at the beginning of 1990s, some negative effect of acidifying species could have been traced when their values were close to the highest over the entire considered period. Over these years, decrease in tree ring widths was detected in 3 of 4 beech stands. On the other hand, the main reason of reduced tree ring width in 2006 could have not been the effect of air concentrations and deposition of accidifying species, because in that year their levels decreased to the lowest values.

### 3.4. Concentrations of Surface Ozone, Their Changes, and Effect on Beech Tree Radial Increment

In contrast to reduction of sulphur emission, the continuing rise in the emissions of precursor substances (VOC, NO, and NO_2_) [48, 49] resulted in a rise in ozone (O_3_) concentrations [50, 51] which as a phytotoxic pollutant and an agent of climate change, recently is considered to be the most important air pollutant for forests [52] and first of all for beech stands [50]. Surface ozone concentrations are expected to substantially increase during Summer time in future climate conditions [52]. Relationship between summertime temperature and surface ozone concentrations is well established [53]. Recent changes in surface temperature should lead to increase in biogenic emissions of isoprene, a biogenic precursor of ozone. This process, which promotes an increase in surface ozone concentrations over southern and central Europe [54], should increase ozone concentrations in Lithuanian as well. However data obtained from Preila EMEP station since 1980 confirm this statement only partially.

**Figure 7:**
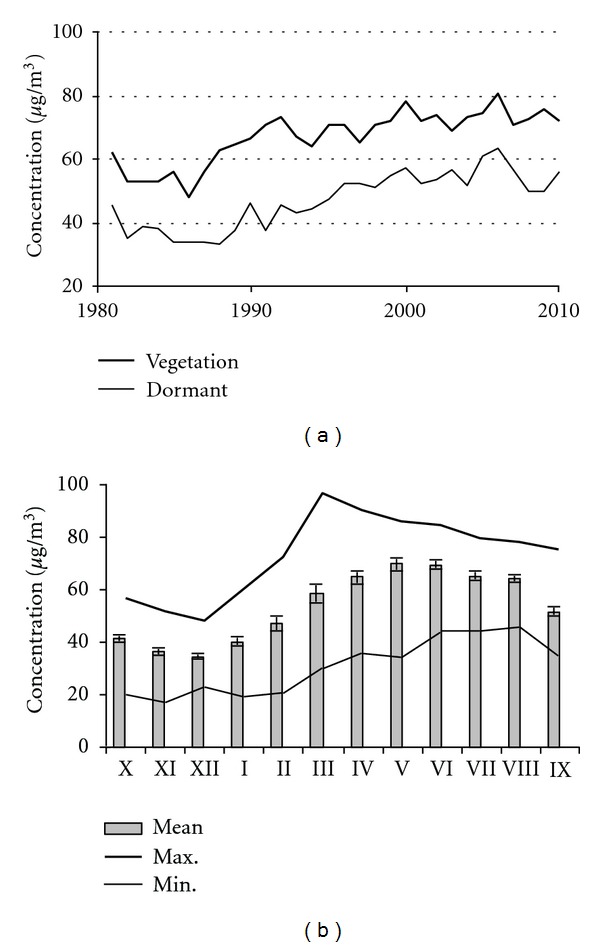
Variation in seasonal and monthly surface ozone concentrations at Preila EMEP station.

The lowest values of ozone concentrations were registered at the beginning of the considered period from 1982 to 1986 (Figure 7). Afterwards ozone concentration started to increase both over the dormant and vegetation period by 0.9 *μ*g/m^3^ and 1.3 *μ*g/m^3^ per year, respectively (*P* < 0.05), confirming predictions of its changes. This process continued until 2006, when mean values of monthly ozone concentration over the vegetation period exceeded 80 mg/m^3^ and over the dormant period 63 mg/m^3^. It was the year when ozone concentrations reached the highest values over the entire monitored period. Over the last 5-year period (2006–2010) ozone concentrations were stable or in contrast to all prognoses started demonstrating a trend towards decreasing by −1.14 *μ*g/m^3^ over vegetation and −2.22 *μ*g/m^3^ over dormant period (*P* > 0.05). These variations in surface ozone concentrations were rather similar to variation in beech tree ring width series. Over the last 5-year period beech tree stem radial increment demonstrated a stable trend or a trend towards increasing.

Variation in mean monthly ozone concentration revealed that ozone concentration reached the highest values mainly on May and June. Despite this, the highest mean monthly value was detected in March, when mean monthly concentration reached 100 *μ*g/m^3^ in 1996. The highest lowest mean monthly values were observed in the period from June through August, when mean monthly concentration did not decrease to less than 40 *μ*g/m^3^. These data revealed that most acceptable period for ozone injuries could be from March to September, and it was taken into account analyzing possible effect of ozone on beech growth.

Since the beginning of this century O_3_ has been recognized as a major phytotoxic agent not only in North America and Southern Europe, but also in Central and Northern Europe including Lithuania [21]. Ozone can cause a wide range of symptoms and one of them is reduction in photosynthesis [55–57], what can result in ultimate reduction of growth and productivity of a tree [50, 58–60]. To detect possible effect of ozone concentration on beech tree radial growth at the initial stage of investigation, simple correlation analysis of the monthly air concentration of ozone and the residual of beech tree ring width when the effect of meteorology was accounted for was performed.

**Figure 8:**
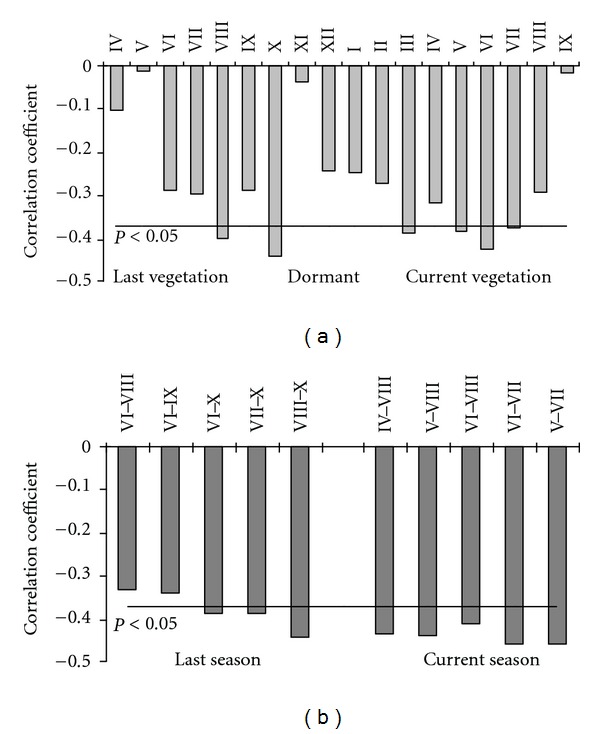
Relationships between the residual of beech tree ring width indices and monthly and seasonal mean concentrations of surface ozone over 30-year period.

Obtained data revealed that the effect of higher concentrations of surface ozone of May through July when growth intensity is highest, resulted in reduction of the beech tree radial increment (Figure 8). Climate change parameters that lead to a longer growing season increase the exposure of tree to ozone injuries [61]. This could have resulted in additional significant effect of ozone concentration of August–October months of the previous year, most probably through the effect on acceleration of leaf senescence and reduction in leaf life span [62–64], which induced early senescence [63], and disruption in carbohydrate allocation patterns [56, 58, 65], which could have altered tree defensive function and resistance to early frost and cold Winter injuries [66].

Aggregated data revealed that mean monthly concentrations over the period from August through October of the previous year and from May through July of the current are key parameters affecting beech tree radial growth (Figure 8). These periods were used in evaluation of ozone effect significance explaining variation in residuals of beech tree ring indices when the effect of meteorological parameters was accounted for by means of multiple regression equation:

(3) $$\begin{array}{cc} Zr_{\text{residual}} & =0.981-0.00817\;\times {{\text{O}}_{3}}_{\left(\text{VIII}-\text{X}\right)}-0.00818\\ & \;\times {{\text{O}}_{3}}_{\left(\text{V}-\text{VII}\right)};\;R²=\;0.238;\;P<0.025, \end{array}\;$$

where: *Zr*_residual_: residual part of beech tree ring width indices; mean concentration of surface ozone:  O_3__(VIII−X)_: over August–October months of previous year; O_3__(V−VII)_: May–July of the current year.

Multiregression analysis revealed that mean monthly concentration of surface ozone over August–October months of the previous year and over May–July of the current year explained up to 24% variation in residual part of beech tree ring width indices when the effect of meteorology was accounted for. This statement agreed well with the data obtained in more southern part of Europe, where up to 25% reduction in beech tree radial increment in areas of elevated ozone concentrations was detected [12, 46, 67].

### 3.5. Integrated Effect of Environmental Factors on Beech Growth and Prognosis

Effects of O_3_ exposure have to be evaluated in the context of changing climate, that is, increasing temperature, changes in water availability, increased available nitrogen due to elevated levels of N deposition, and many other factors [47]. Synergistic O_3_ effects with high temperature and moisture stress are well known [68]. However, the statement that the effect of O_3_ and drought might counterbalance each other [17] is more significant when investigating phytotoxic O_3_ effect on trees. Closed stomata protect foliage from the uptake of high O_3_ concentrations into leaves, which is typical of periods characterized by high temperature and moisture stress. Most likely, therefore, the O_3_ effect in northern latitudes (where moisture stress is less frequent) often leads to plants becoming more susceptible to injury than in southern areas, despite the increase in O_3_ concentrations from north to south [50, 69, 70], which proves recently presented data on the phytotoxic effect of regional O_3_ concentration on forests in central and northern Europe [21, 46, 71, 72]. In this study, we attempted to investigate possible effect of meteorological parameters and acidifying species as well as surface ozone on beech tree stem radial growth and quantify O_3_ contributions to the integrated impact of these factors. To meet this aim, mean concentration of ozone over the detected periods and annual values of acidifying species were included into (1). Only ozone values remained at the significant level. Effect of acidifying species was not significant what resulted in their elimination from the model. Developed model is as follows:

(4) $$\begin{array}{cc} Zr_{\text{index}} & =\;3.184-0.0705\times Tm_{\left(\mathrm{VI}-\text{VIII}\right)}-0.0398\;\\ & \;\times {Tm^{\prime }}_{\left(\mathrm{VI}-\text{VIII}\right)}+\;0.0328\times Tm_{\left(\text{X}-\text{XII}\right)}\;+\;0.0005\;\;\\ & \;\times \;\left[Tm\times {\text{Pr}}_{\left(\text{III}-\text{V}\right)}\right]-\;0.0163\times {{\text{O}}_{3}}_{\left(\text{V}-\text{VII}\right)}\\ & \;-0.0123\;\times {{\text{O}}_{3}}_{\left(\text{VIII}-\text{X}\right)};\;R²=\;0.749;\;P<0.0001, \end{array}\;$$

where: *Tm*: mean air temperature of current year; *Tm*′: of previous year; Pr: precipitation amount;  *Tm* × Pr: multiplication of temperature and precipitation amount; (*in brackets*): the selected period in months;  O_3_: ozone concentration, (*μ*g/m^3^).

Significance of ozone concentration explaining variation in beech tree ring indices in the integrated effect with meteorology on beech tree growth made 18%, which allows concluding that surface ozone is among key factors for beech growth outside their north-eastern distribution range contributing to reduction of their resistance to heat and lack of the humidity over the vegetation period, and cold over the winter months. These results confirmed the statement that climate change increases sensitivity of beech trees in boreal areas and decreases in temperate areas due to temperature and water stress [61].

**Figure 9:**
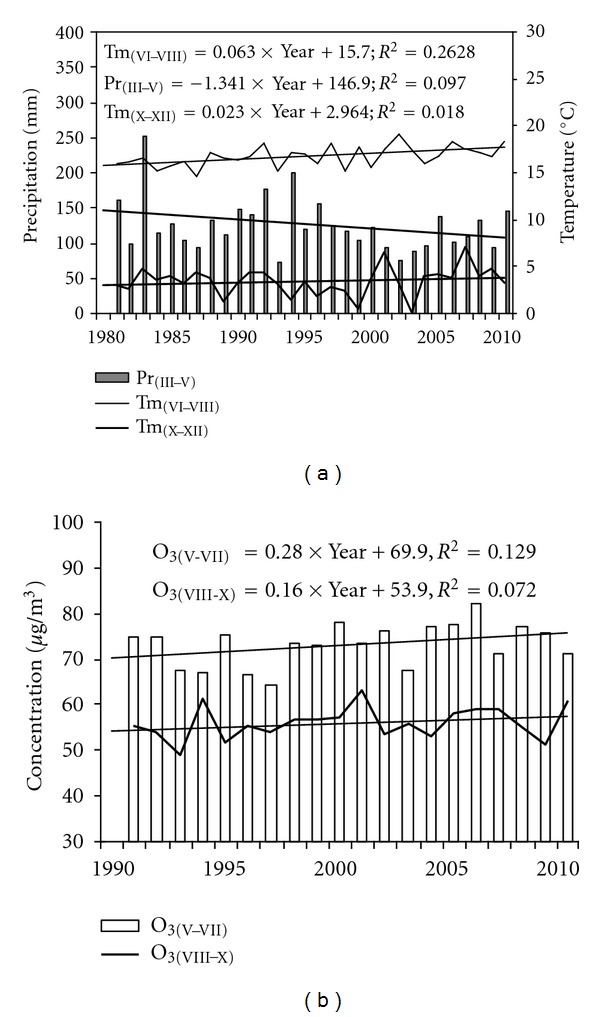
Variation of the key parameters limiting the growth of beech trees in Lithuania.

Analysis of temporal variation of key environmental parameters resulting in beech tree growth intensity revealed that in future the variation in mean air temperature of June–August, which shows tendencies towards increasing and variation in precipitation amount over March–May months, which shows tendencies towards decreasing should result in reduction of beech tree radial increment. Contrary to this, variation in mean temperature of October through December which shows a tendency towards decreasing should reduce the possibilities of tree injuries by frost, meanwhile quite stabile variation in ozone concentrations should not contributed to reduction of tree increment and increase sensitivity of beech trees to drought and frost. Finally, based on the detected significance of variation in key parameters of beech growth, that is, increase in temperature over the June–August and lack of the humidity in spring; it is rather problematic to expect better environmental conditions for beech tree growth outside their natural north-eastern distribution range in central part of Lithuania. Despite this European beech growing with Scots pine increases total stand volume up to 30–50%, which makes this species very acceptable in forestry practice (Figure 9).

## 4. Conclusions

European beech (*Fagus sylvatica *L.) is one of the most widespread and thoroughly investigated European tree species. Despite this, a limited number of studies have examined mature beech tree growth outside their natural distribution range in relation to meteorological parameters and air pollution. The obtained results revealed that hot Summers, cold dormant, and dry and cold first-half of vegetation period resulted in beech tree growth reduction. These meteorological parameters explained more than 57% variation in beech tree ring widths. 30-year data on acidifying species and surface ozone obtained from Preila EMEP station showed that acidifying species in the air and wet deposition had no significant effect on variation in beech tree ring width data series. Only surface ozone, mean monthly concentrations of which, often were below phytotoxic levels was among key factors resulting in beech stand productivity at northern latitude of their pervasion. Phytotoxic effect of this pollutant increased explanation rate of beech tree ring variation by 18%, that is, up to 75%. Therefore under recent global changes, it is rather problematic to expect better environmental conditions for beech tree growth outside their natural north-eastern distribution range. Despite this European beech growing with Scots pine increases total stand volume up to 30–50%, which makes this species very acceptable in forestry practice, while warmer dormant periods reduce the possibility to die due to frost.

## Figures and Tables

**Table 1:** Main dendrometric characteristics of the monitored stands.

Site	Level	Tree species	Number of trees	Age	Diameter	Height	Stocking	Sum of basal area	Volume	Defoliation
			unit/ha	year	cm	m		m^2^/ha	m^3^/ha	%
B 1	I	B	440	75	25.1	25.8	0.50	25.0	295.8	9.1
B 1							**0.50**	**25.0**	**295.8**	
B 2	I	Q	280	105	38.1	29.5	0.70	33.6	447.2	34.1
B 2	II	B	710	70	16.3	23.4	0.30	16.2	180.2	13.1
B 2							**1.0**	**49.8**	**627.4**	
B 3	I	P	205	125	43.1	33.2	0.55	31.5	467.5	22.6
B 3	II	B	255	70	25.5	30.3	0.25	14.5	199.1	11.7
B 3	II	A	75		31.1	28.1	0.10	6.2	81.5	22.6
B 3	II	Ot. sp.	75		30.9	30.2	0.10	5.6	70.5	22.6
B 3							**1.0**	**57.8**	**818.6**	
B 4	I	P	840	70	16.1	24.3	0.60	19.1	220.6	14.3
B 4	II	B	720	70	17.6	25.6	0.40	19.8	237.7	12.6
B 4							**1.0**	**39.0**	**458.3**	

Note: Tree species: B: *Fagus sylvatica*; P: *Pinus sylvestris*; Q: *Quercus robur*; A: *Acer platanoides*, Ot.sp.: other species: *Fraximus excelsior*, *Tilia cordata*, *Quercus robur,* and* Picea abies*.
